# Trajectories and influencing factors of cognitive function and physical disability in Chinese older people

**DOI:** 10.3389/fpubh.2024.1380657

**Published:** 2024-07-04

**Authors:** Shuyuan Cheng, Rong Yin, Kunpeng Wu, Qiong Wang, Hui Zhang, Li Ling, Wen Chen, Leiyu Shi

**Affiliations:** ^1^International Cooperation and Exchange Department, The First Affiliated Hospital of Sun Yat-sen University, Guangzhou, China; ^2^Health Policy and Management Department, Bloomberg School of Public Health, Johns Hopkins University, Baltimore, MD, United States; ^3^Department of Medical Statistics, School of Public Health, Sun Yat-sen University, Guangzhou, China; ^4^Department of Epidemiology, School of Public Health, Sun Yat-sen University, Guangzhou, China; ^5^Department of Health Policy and Management, School of Public Health, Sun Yat-sen University, Guangzhou, China; ^6^Department of Health Policy and Management, Bloomberg School of Public Health, Johns Hopkins University, Baltimore, MD, United States

**Keywords:** disability, cognitive function, aging, development trajectory, influencing factors

## Abstract

**Introduction:**

Dementia and physical disability are serious problems faced by the aging population, and their occurrence and development interact.

**Methods:**

Based on data from a national cohort of Chinese people aged 60 years and above from the China Health and Retirement Longitudinal Survey from 2011 to 2018, we applied the group-based trajectory model to identify the heterogeneous trajectories of cognitive function and physical disability in participants with different physical disability levels. Next, multinomial logistic regression models were used to explore the factors affecting these trajectories.

**Results:**

The cognitive function trajectories of the Chinese older people could be divided into three characteristic groups: those who maintained the highest baseline level of cognitive function, those with a moderate baseline cognitive function and dramatic progression, and those with the worst baseline cognitive function and rapid–slow–rapid progression. The disability trajectories also fell into three characteristic groups: a consistently low baseline disability level, a low initial disability level with rapid development, and a high baseline disability level with rapid development. Compared with those free of physical disability at baseline, a greater proportion of participants who had physical disability at baseline experienced rapid cognitive deterioration. Education, income, type of medical insurance, gender, and marital status were instrumental in the progression of disability and cognitive decline in the participants.

**Discussion:**

We suggest that the Chinese government, focusing on the central and western regions and rural areas, should develop education for the older people and increase their level of economic security to slow the rate of cognitive decline and disability among this age group. These could become important measures to cope with population aging.

## Introduction

Global aging is increasing, with one sixth of the world’s population being over the age of 60 in 2023 (1). Several studies have shown that the prevalence of dementia and physical disability in the older people increases with age (2, 3). The number of people with dementia worldwide is expected to increase from 57.4 million in 2019 to 152.8 million in 2050 (4). In China, there were approximately 16.25 million people with dementia in 2020, and that number is expected to approximately triple to 48.98 million by 2050 (5). Notably, the proportion of older people with disabilities among the total number of persons with disabilities in China is projected to exceed 57% in 2030, and will further increase to over 70% by 2050 if no preventive or control measures are taken (6). Meanwhile, increased longevity has led to a sustained increase in the number of years lived with physical disability, which has in turn increased the financial burden in later life for both disabled individuals and society (7, 8). Cognitive function and physical disability present major challenges to healthy aging today (9). Understanding the progression of cognitive degeneration and physical disability can contribute to the formulation of preventive or control measures.

During the progression from normal cognition to dementia, there is an intermediate stage of ‘mild cognitive impairment’ (10), which does not necessarily get progressively worse and may be reversible, according to previous research (11, 12). Existing studies have explored the trajectory of cognitive degeneration in the older people, but their findings on the typology of these trajectories have been inconsistent. Some studies, including three in China, have reported between three and six trajectories of cognitive degeneration among the older people (13–24). Specifically, some studies in the United States (US), the United Kingdom (UK), Japan, and China have shown that the higher the baseline cognitive level of older people, the slower their future decline in cognitive function. However, two other studies in China found that the older population with the most stable maintenance of cognitive function comprised people with a moderate baseline cognitive function (19, 20).

There is similar variation in studies on the trajectories of physical disability in the older people. Regarding the progression of mobility degeneration, the existence of a reversible trajectory among older adults has only been supported by two studies, both conducted in the Netherlands (25, 26). Nusselder et al. identified two groups of Dutch persons aged 15–74 years with reversible trajectories (26). One group was characterized by initial mild disability and gradual functional improvement, while those in the other group were moderately disabled at baseline with partial recovery in the subsequent months. In Gardeniers et al., the recovery of physical function was observed only among Dutch men aged 75 years and above, not their female counterparts (25). Some studies have identified between three and nine trajectories of physical disability in the older people (26, 27). Additionally, many researchers in the UK, the US, the Netherlands, and China have identified two groups with opposite patterns of mobility degeneration: those suffering the severest disability in the beginning, progressing most rapidly and reaching the worst status during the follow-up period; and those with no or little disability initially, then persistent low levels of physical disability over a period of years (25, 28–30). However, another study in China in 2015 showed that older people with a low level of disability (1 item of disability) at baseline later experienced the highest level of disability (nearly 10 items of disability) due to the rapid progression of their disability over the following decade (31).

Most previous studies have focused on the progression of physical disability and cognitive function separately. However, it has been shown that the two mutually affect each other (32, 33) and that there is a possibility of co-morbidity (34). Therefore, it is relevant to study the covarying trajectory of cognitive function and disability. At present, only five studies, all conducted in the US, have studied both cognitive function and physical disability, and they have found that the trajectories of disability vary with the state of cognitive function (27, 35–38). For example, a 2016 study by Tolea et al. involving US adults aged 50 and above found that older people with dementia experienced a decline in mobility (27). However, the current evidence on the trajectory of cognitive function in people with different disability levels remains limited. To date, no published studies in China have explored the trajectories of cognitive function and disability together.

Studies have shown that cognitive function trajectories are associated with genetics, the presence of other diseases, lifestyles, and socioeconomic status. Existing evidence suggests that the AOPEε4 gene may accelerate cognitive decline (39–41). Mental disorders, cardiovascular disease, and other chronic diseases have been identified as major contributors to accelerating declines in both cognition and mobility (42–44). In terms of lifestyle, poor nutrition and physical inactivity worsen both physical disability and cognitive function (45, 46). Previous studies have also revealed that education, income, occupation, and residential surroundings can differentially affect the rate of cognitive and disability decline in the older people (47–49). However, studies among China’s older population have mainly focused on the effects of factors such as gender, disease, and education (50); there is a lack of comprehensive analysis of multiple factors.

This study was designed to reveal the trajectories of both cognitive function and physical disability among older people in China, and to identify the factors contributing to them, utilizing data from the China Health and Retirement Longitudinal Survey (CHARLS) (51), a prospective study with a national cohort, spanning from 2011 to 2018. The results have important implications for developing practical strategies to address current issues facing the aging Chinese population.

## Methods

### Participants and setting

**Figure 1:**
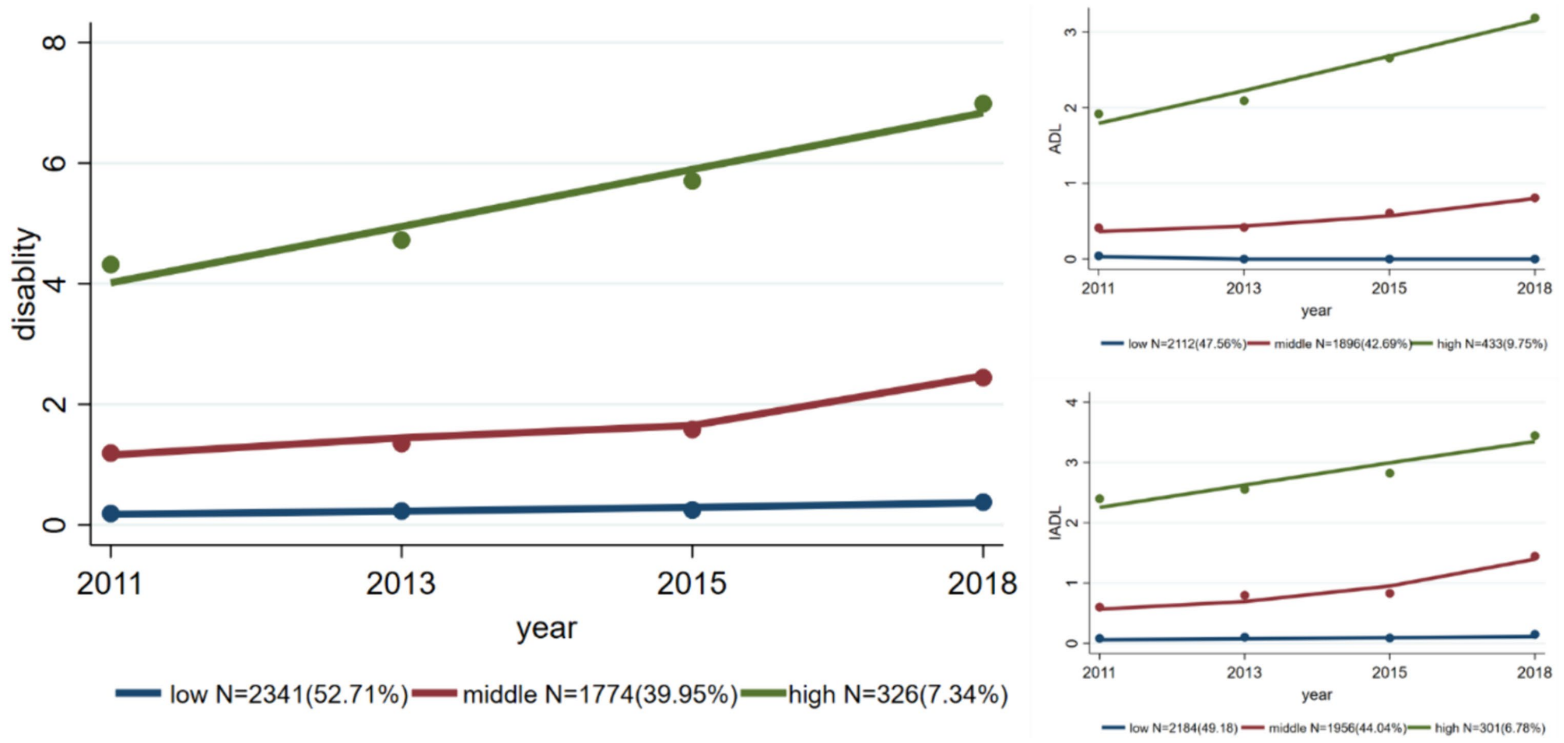
Trajectories of overall physical disability and of disability assessed by ADL and IADL separately.

CHARLS survey data from 2011, 2013, 2015, and 2018 were used. A total of 7,961 persons aged 60 and above were included in this study from the 2011 baseline of 17,708 respondents. We further excluded those who were lost to follow up (1,094 in 2013, 795 in 2015, and 892 in 2018) and 469 persons whose test results for cognitive function and physical disability were missing, resulting in a final study population sample size of 4,441 (see Figure 1).

### Outcome measurements

#### Physical disability

Physical disability was assessed using scales of activities of daily living (ADL) and instrumental activities of daily living (IADL). Six questions (on bathing, dressing, eating, getting up/out of bed, toileting, and bowel control) were included in the ADL scale and five questions (on chores, cooking, shopping, phone calls, and medication) were included in the IADL scale. In the original questionnaire (52, 53), the responses to each question were divided into four levels (‘no difficulty’, ‘difficult but still able to complete’, ‘difficult and need help’, ‘unable to complete’). In this study, any level of difficulty was defined as the presence of disability, and ‘no difficulty’ was defined as the absence of disability. This approach generated a disability score between 0 and 11, with a higher number indicating more limitation in daily activities.

#### Cognitive function

Two components of cognitive function were measured: episodic memory and psychiatric status (53–56). Reverse scoring was adopted, with a higher score representing poorer cognitive function. Episodic memory was measured by testing the respondents’ ability to recall words, with 10 words tested for immediate memory and 10 for delayed memory. A wrong answer scored 1 point and a correct answer scored 0 points, yielding a total score ranging from 0 to 20 points, with higher scores representing worse episodic memory (57). Psychiatric status was measured by the time orientation, numeracy, and constructive drawing abilities of the survey respondents (58). The time orientation measurement asked the respondents the year, month, day, season, and day of the week at the time of the survey, with a wrong answer scoring 1 point and a correct answer scoring 0 points. Numeracy was measured by asking the respondents to calculate 100 minus 7 five consecutive times, with each wrong answer scoring 1 point and each correct answer 0 points. Constructive drawing ability was measured by asking the respondents to draw from a graphic shown by the investigator, with 0 points being assigned for correct drawings and 1 point for each error. The total cognitive function scores ranged from 0 to 31, with higher scores representing poorer cognitive function.

#### Covariates

The covariate data collected were age (60–69 years, 70–79 years, ≥80 years), gender (male, female), education (illiterate, elementary school, junior high school, high school and above), marital status (no partner, partnered), type of residence (rural, urban), region (eastern, central, western), annual household income (RMB0–9,999, RMB10,000–49,999, ≥RMB50,000), and type of medical insurance (Urban Employee Basic Medical Insurance, Urban and Rural Resident Basic Medical Insurance, Other).

### Statistical analysis

The physical disability scores, including the total scores and scores for ADL and IADL, in the four survey waves were described by *n* (%). Medians (interquartile ranges) were used to describe the cognitive function scores, including total scores and the scores for episodic memory and psychiatric status.

Group-based trajectory modeling (GBTM) (59, 60) was employed to identify similarities in the developmental trajectories of cognitive function and physical disability among the participants. Referring to previous studies, most of which have identified three groups of trajectories (17–21), we *a priori* set three as the number of groups. The respondents were categorized into the groups to which the progression patterns of their three trajectories had the maximum predicted probability of belonging. Next, we used chi-square tests to test the differences in baseline characteristics among the three groups. GBTM was conducted separately for the total disability scores, scores for ADL and IADL, total cognitive function scores, scores for episodic memory, and scores for psychiatric status.

For trajectories considering both physical disability and cognitive function simultaneously, we first divided all the participants into two subgroups based on the disability scores: absence of disability (‘non-disabled’; disability score = 0) and presence of disability (‘disabled’; disability score ≥ 1). The cognitive function trajectory was then identified separately for the two subgroups using GBTM. In line with the aforementioned analysis, we *a priori* set three groups for GBTM and employed chi-square tests to analyze the differences in baseline characteristics.

A multinomial logistic regression model was used to analyze the factors influencing the trajectories of disability and cognitive function separately. The covariates were age, gender, education, marital status, type of residence, region, annual household income, and type of medical insurance. Due to limited sample size, we did not use a multinomial logistic regression model to identify the factors influencing the trajectories of cognitive function for subgroups with the presence and absence of disability. In addition, two sensitivity analyses were conducted to examine (1) the potential interactions of baseline cognitive function/physical disability score with other characteristics on physical disability and cognitive function trajectory, respectively, and (2) the potential moderation of hypertension on physical disability and cognitive function trajectory. Because, hypertension is important as comorbidities need to be taken in account as it is very frequent and is a risk factor for cognitive impairment and physical disability.

The software packages R 4.2.3 and Stata 17.0 were used for the statistical analyses, and the two-tailed test level of α was 0.05.

All waves of CHARLS were approved by the Institutional Review Board (IRB) of Peking University (IRB00001052-11015 for household survey and IRB00001052-11014 for biomarker collection). All participants signed informed consent.

## Results

### Baseline characteristics of the surveyed population

As shown in Table 1, the majority of the participants were aged 60–69 years (71.8%) at baseline, and approximately half were women (51.3%). Many were illiterate (34.8%) or had only a primary school education level (46.8%). A small number of the participants had no partner (18.1%). Although a minority were from urban areas (17.6%), the regional distribution was relatively even (37.7% from eastern China, 36.2% from central China, and 26.1% from western China). The annual household income of more than half of the participants was less than RMB10,000 (58.9%), and the main type of medical insurance was medical insurance for urban and rural residents (80.5%).

**Table 1:** Baseline characteristics of the participants.

Baseline characteristics	Overall	Disability trajectory group			*P*	Cognitive function trajectory group			*P*
		Low	Middle	High		Low	Middle	High	
*N*	4,441	2,341	1,774	326		1,546	1,631	1,264	
Age (years), *N* (%)					<0.001				<0.001
60–69	3,190 (71.8)	1,876 (80.1)	1,139 (64.2)	175 (53.7)		1,288 (83.3)	1,209 (74.1)	693 (54.8)	
70–79	1,099 (24.7)	427 (18.2)	546 (30.8)	126 (38.7)		241 (15.6)	389 (23.9)	469 (37.1)	
80–	152 (3.4)	38 (1.6)	89 (5.0)	25 (7.7)		17 (1.1)	33 (2.0)	102 (8.1)	
Sex, *N* (%)					<0.001				<0.001
Male	2,164 (48.7)	1,375 (58.7)	672 (37.9)	117 (35.9)		967 (62.5)	833 (51.1)	364 (28.8)	
Female	2,277 (51.3)	966 (41.3)	1,102 (62.1)	209 (64.1)		579 (37.5)	798 (48.9)	900 (71.2)	
Education, *N* (%)					<0.001				<0.001
No formal education	1,547 (34.8)	536 (22.9)	838 (47.2)	173 (53.1)		82 (5.3)	515 (31.6)	950 (75.2)	
Primary school	2,080 (46.8)	1,204 (51.4)	752 (42.4)	124 (38.0)		838 (54.2)	949 (58.2)	293 (23.2)	
Junior middle school	542 (12.2)	397 (17.0)	123 (6.9)	22 (6.7)		394 (25.5)	130 (8.0)	18 (1.4)	
Middle school or above	272 (6.1)	204 (8.7)	61 (3.4)	7 (2.1)		232 (15.0)	37 (2.3)	3 (0.2)	
Marital status, *N* (%)					<0.001				<0.001
Partnered	3,638 (81.9)	2,020 (86.3)	1,369 (77.2)	249 (76.4)		1,372 (88.7)	1,356 (83.1)	910 (72.0)	
Single	803 (18.1)	321 (13.7)	405 (22.8)	77 (23.6)		174 (11.3)	275 (16.9)	354 (28.0)	
Residence status, *N* (%)					<0.001				<0.001
Rural	3,661 (82.4)	1,811 (77.4)	1,561 (88.0)	289 (88.7)		1,046 (67.7)	1,435 (88.0)	1,180 (93.4)	
Urban	780 (17.6)	530 (22.6)	213 (12.0)	37 (11.3)		500 (32.3)	196 (12.0)	84 (6.6)	
Geographic distribution, *N* (%)					<0.001				<0.001
Eastern China	1,675 (37.7)	1,002 (42.8)	593 (33.4)	80 (24.5)		645 (41.7)	601 (36.8)	429 (33.9)	
Central China	1,607 (36.2)	811 (34.6)	657 (37.0)	139 (42.6)		602 (38.9)	589 (36.1)	416 (32.9)	
Western China	1,159 (26.1)	528 (22.6)	524 (29.5)	107 (32.8)		299 (19.3)	441 (27.0)	419 (33.1)	
Household family income per year (RMB), *N* (%)					<0.001				<0.001
0–9,999	2,614 (58.9)	1,222 (52.2)	1,168 (65.8)	224 (68.7)		745 (48.2)	991 (60.8)	878 (69.5)	
10,000–49,999	1,467 (33.0)	881 (37.6)	498 (28.1)	88 (27.0)		642 (41.5)	526 (32.3)	299 (23.7)	
50,000+	360 (8.1)	238 (10.2)	108 (6.1)	14 (4.3)		159 (10.3)	114 (7.0)	87 (6.9)	
Medical insurance, *N* (%)					<0.001				<0.001
Urban and Rural Resident Basic Medical Insurance	3,577 (80.5)	1,779 (76.0)	1,516 (85.5)	282 (86.5)		1,044 (67.5)	1,401 (85.9)	1,132 (89.6)	
Urban Employee Basic Medical Insurance	436 (9.8)	322 (13.8)	99 (5.6)	15 (4.6)		327 (21.2)	91 (5.6)	18 (1.4)	
Other	428 (9.6)	240 (10.3)	159 (9.0)	29 (8.9)		175 (11.3)	139 (8.5)	114 (9.0)	

### Physical disability and cognitive function across four survey waves

Table 2 shows the disability and cognitive function scores across the four survey waves. The proportion of participants free of physical disability gradually decreased from the 2011 to 2018 survey waves, with percentages of 65.9, 57.4, 51.0, and 46.6%. Few participants had a disability score above 5 points (<5%). The same trend was observed for IADL measurements, with the proportion of participants without physical disability being 74.6, 64.3, 59.7, and 53.5% for each survey wave. Compared with that of IADL disabilities, the prevalence of ADL disabilities was slightly lower, with the corresponding percentages of disability-free participants being 78.6, 78.1, 71.6, and 68.7%, respectively.

**Table 2:** Disability and cognitive function scores across the four survey waves (*N* = 4,441).

	2011	2013	2015	2018
Disability score^a^, *N* (%)				
0	2,927 (65.9)	2,548 (57.4)	2,266 (51.0)	2,071 (46.6)
1	632 (14.2)	918 (20.7)	956 (21.5)	863 (19.4)
2	333 (7.5)	361 (8.1)	417 (9.4)	410 (9.2)
3	162 (3.6)	206 (4.6)	230 (5.2)	270 (6.1)
4	100 (2.3)	131 (2.9)	184 (4.1)	203 (4.6)
5	87 (2.0)	80 (1.8)	114 (2.6)	153 (3.4)
6	58 (1.3)	63 (1.4)	85 (1.9)	135 (3.0)
7	53 (1.2)	40 (0.9)	56 (1.3)	88 (2.0)
8	26 (0.6)	41 (0.9)	58 (1.3)	69 (1.6)
9	35 (0.8)	25 (0.6)	36 (0.8)	64 (1.4)
10	18 (0.4)	20 (0.5)	23 (0.5)	60 (1.4)
11	10 (0.2)	8 (0.2)	16 (0.4)	55 (1.2)
ADL score, *N* (%)				
0	3,491 (78.6)	3,468 (78.1)	3,179 (71.6)	3,053 (68.7)
1	501 (11.3)	505 (11.4)	613 (13.8)	591 (13.3)
2	203 (4.6)	228 (5.1)	299 (6.7)	301 (6.8)
3	104 (2.3)	103 (2.3)	157 (3.5)	181 (4.1)
4	68 (1.5)	70 (1.6)	99 (2.2)	125 (2.8)
5	53 (1.2)	51 (1.1)	62 (1.4)	103 (2.3)
6	21 (0.5)	16 (0.4)	32 (0.7)	87 (2.0)
IADL score, *N* (%)				
0	3,313 (74.6)	2,854 (64.3)	2,649 (59.7)	2,375 (53.5)
1	522 (11.8)	920 (20.7)	985 (22.2)	899 (20.2)
2	285 (6.4)	332 (7.5)	381 (8.58)	437 (9.8)
3	166 (3.7)	189 (4.3)	221 (4.98)	321 (7.2)
4	96 (2.2)	101 (2.3)	133 (2.9)	225 (5.1)
5	59 (1.3)	45 (1.0)	72 (1.6)	184 (4.1)
Cognitive function score (median [interquartile range])	18.00 [14.00, 23.00]	19.00 [14.00, 24.00]	19.00 [15.00, 24.00]	22.00 [15.00, 28.00]
Episodic memory score (median [interquartile range])	14.00 [11.00, 17.00]	14.00 [11.00, 17.00]	15.00 [12.00, 17.00]	15.00 [12.00, 20.00]
Psychiatric status score (median [interquartile range])	4.00 [1.00, 7.00]	5.00 [1.00, 8.00]	5.00 [2.00, 8.00]	6.00 [3.00, 9.00]

^a^Disability score is the sum of ADL and IADL scores.

The cognitive function of the participants also deteriorated over the four waves of the survey, with the median cognitive function score increasing from 18 points in the 2011 wave to 19 points in 2013 and 2015, and 22 points in the 2018 wave. As an element of cognitive function, the psychiatric status worsened, with the median score rising from 4 points in 2011 to 5 points in 2013 and 2015, and 6 points in the 2018 wave. Meanwhile, deterioration in episodic memory occurred more slowly, with the median score increasing from 14 points in 2011 to 15 points in the 2018 wave.

### Trajectories of disability and cognitive function

Three disability trajectories are shown in Figure 1. The trajectory with the consistently lowest level of disability was denoted the LOW group. This group (*N* = 2,341) maintained a low disability level across the four survey waves and accounted for 52.71% of the total participants. The participants (*N* = 1,774, 39.95%) with a somewhat higher disability level at baseline were categorized as the MIDDLE group. The disability score in this group started at a relatively low level, increased slowly from the 2011 to the 2015 wave, but then increased more rapidly in later years. The group with the highest baseline disability level, the HIGH group, contained the smallest number of participants (*N* = 326, 7.34%). The disability score of this group increased rapidly from 4 in 2011 to approximately 7 in the 2018 wave. The trajectories of disability assessed using ADL and IADL showed similar trends (Figure 1).

**Figure 2:**
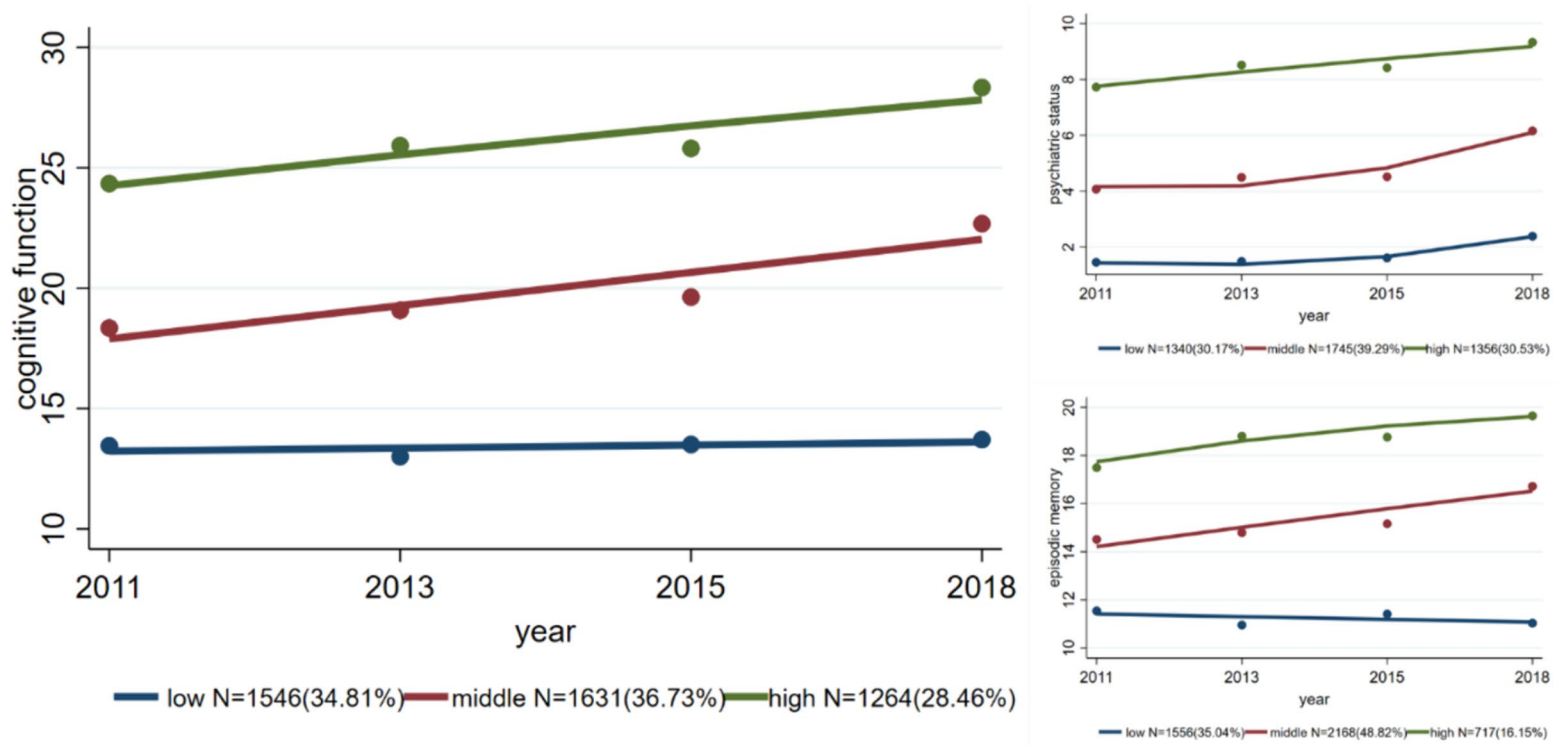
Trajectories of overall cognitive function, psychiatric status, and episodic memory.

Figure 2 shows three cognitive function trajectories. The LOW group started with the best cognitive function and consistently maintained a score of approximately 14 points (out of 20) across the four survey waves. This group accounted for 34.81% of the total participants (*N* = 1,546). The MIDDLE group comprised participants with marked declines in cognitive function. In this group (*N* = 1,631, 36.73%), the cognitive function score was approximately 18 points in the 2011 wave, but dramatically increased to approximately 23 points in the 2018 wave. The HIGH group, i.e., the group with the worst cognitive function, had a baseline score of over 24 points, which rapidly increased to approximately 28 points by the 2018 wave, and represented 28.46% of the total respondents (*N* = 1,264). The trajectories of episodic memory and psychiatric status showed similar trends.

**Figure 3:**
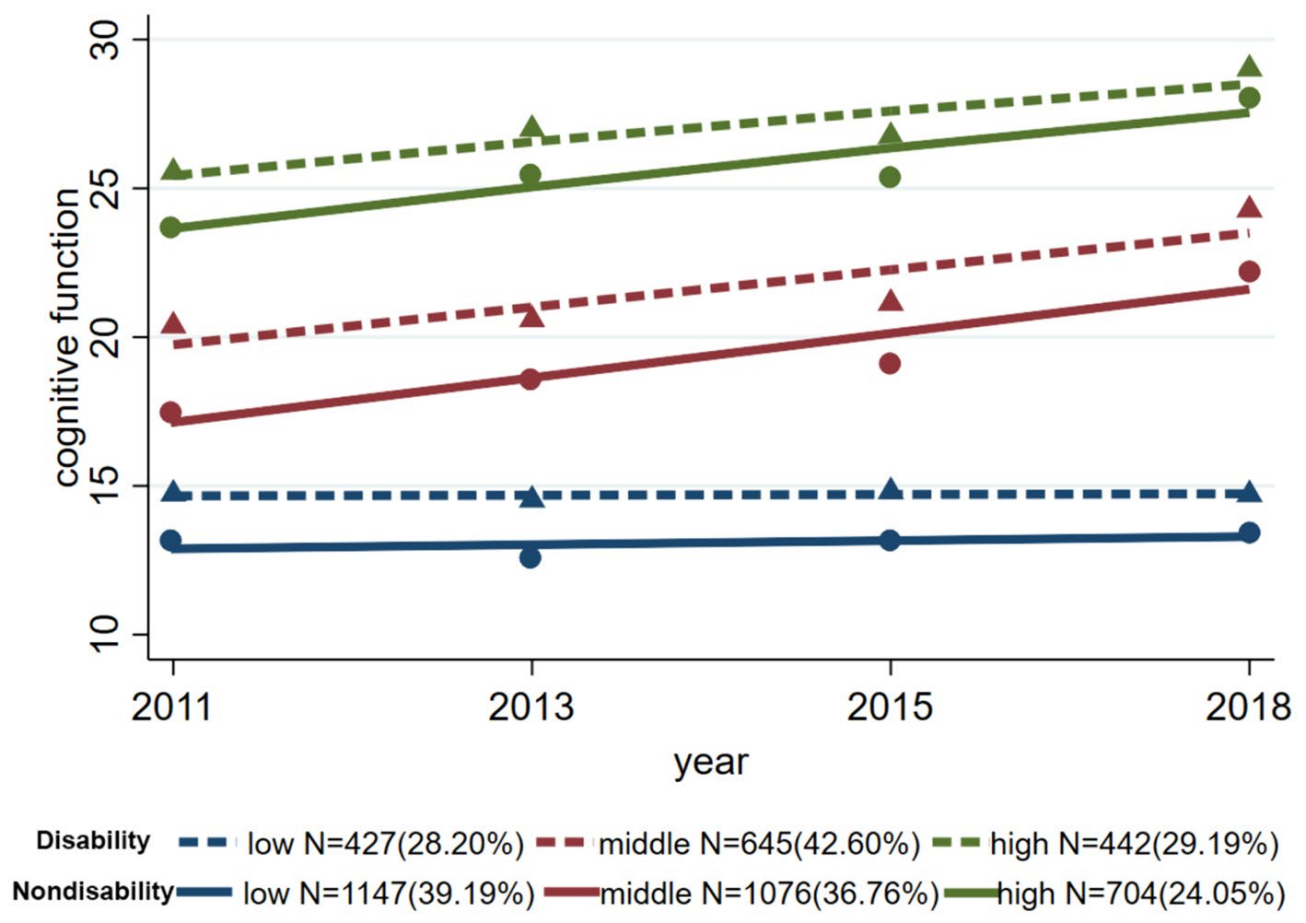
Trajectories of cognitive function in the disabled and non-disabled subgroups.

Figure 3 shows the trajectories of cognitive function in the disabled and non-disabled subgroups. Although the trajectories of the three groups in each subgroup showed the same trend, the cognitive function scores in the disabled group were lower than those in the non-disabled group. In terms of the distribution of participants, the non-disabled subgroup had more participants in the LOW disability trajectory group (*N* = 1,147, 39.19%) than the disabled group did (*N* = 427, 28.20%), but had fewer participants in the MIDDLE (36.76% vs. 42.60%) and HIGH disability trajectory groups (24.05% vs. 29.19%).

### Factors influencing the trajectories of physical disability and cognitive function

**Figure 4:**
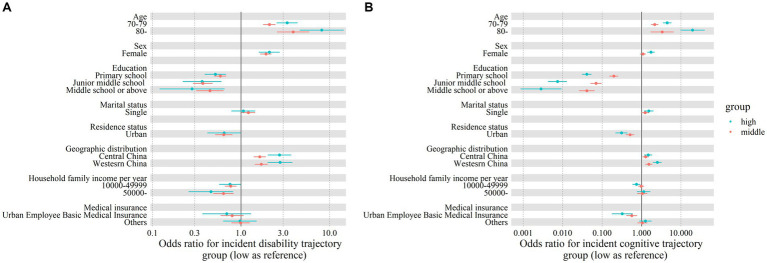
Odds ratios (ORs) and 95% confidence intervals (95% CIs) for factors associated with disability trajectory groups **(A)** and cognitive function trajectory groups **(B)**. Solid dots and error bars, respectively, denote the OR and 95% CI estimates.

The association between various factors and the trajectories of physical disability and cognitive function are shown in Figure 4. Compared with the LOW disability trajectory group, the risk factors for the MIDDLE group were an age of 70–79 years (odds ratio [OR]: 2.089; 95% confidence interval [CI]: 1.778, 2.456) or 80 years and older (3.879; 2.556, 5.887); being female (1.913; 1.654, 2.213); and living in the central (1.628; 1.392, 1.904) and western (1.697; 1.431, 2.012) regions of China. The protective factors were primary school (0.585; 0.501, 0.683), junior high school (0.370; 0.287, 0.477), or high school or above (0.446; 0.313, 0.635) education; urban residence (0.639; 0.510, 0.801); and an annual household income of RMB10,000–49,999 (0.765; 0.658, 0.890) or more than RMB50,000 (0.634; 0.487, 0.825). The risk and protective factors for the LOW and HIGH groups were similar, although the estimated ORs were slightly different.

The factors associated with cognitive function trajectories were similar to those associated with physical disability trajectories. Compared with the LOW cognitive function trajectory group, the risk factors for the MIDDLE group were an age of 70–79 years (OR: 2.154; 95% CI: 1.745, 2.659) or 80 years and older (3.330; 1.697, 6.535); and living in the central (1.267; 1.058, 1.518) or western (1.534; 1.250, 1.812) regions of China. The protective factors for the MIDDLE group were primary school (0.198; 0.152, 0.256), junior high school (0.070; 0.051, 0.097), or high school or above (0.041; 0.026, 0.064) education; urban residence (0.512; 0.400, 0.655); and holding medical insurance for urban workers (0.565; 0.413, 0.775). The risk and protective factors for the LOW and HIGH group were similar, although the estimated ORs were slightly different (Figure 4).

The differences in baseline characteristics between the trajectory groups, for both the disabled and non-disabled subgroups, are shown in Table 3. All baseline characteristics were significantly different among the three trajectories of cognitive function, regardless of disability status.

**Table 3:** Baseline characteristics of the groups in three cognitive function trajectories, by physical disability status.

Baseline characteristics	Cognitive function trajectory group in the disabled subgroup			*P*	Cognitive function trajectory group in the non-disabled subgroup			*P*
	Low	Middle	High		Low	Middle	High	
*N*	427	645	442		1,147	1,076	704	
Age (years), *N* (%)				<0.001				<0.001
60–69	352 (82.4)	449 (69.6)	200 (45.2)		963 (84.0)	805 (74.8)	421 (59.8)	
70–79	67 (15.7)	175 (27.1)	195 (44.1)		175 (15.3)	254 (23.6)	233 (33.1)	
80–	8 (1.9)	21 (3.3)	47 (10.6)		9 (0.8)	17 (1.6)	50 (7.1)	
Sex, *N* (%)				<0.001				<0.001
Male	238 (55.7)	254 (39.4)	112 (25.3)		743 (64.8)	592 (55.0)	225 (32.0)	
Female	189 (44.3)	391 (60.6)	330 (74.7)		404 (35.2)	484 (45.0)	479 (68.0)	
Education, *N* (%)				<0.001				<0.001
No formal education	37 (8.7)	283 (43.9)	362 (81.9)		53 (4.6)	303 (28.2)	509 (72.3)	
Primary school	249 (58.3)	326 (50.5)	77 (17.4)		607 (52.9)	641 (59.6)	180 (25.6)	
Junior middle school	99 (23.2)	29 (4.5)	3 (0.7)		298 (26.0)	100 (9.3)	13 (1.8)	
Middle school or above	42 (9.8)	7 (1.1)	0 (0.0)		189 (16.5)	32 (3.0)	2 (0.3)	
Marital status, *N* (%)				<0.001				<0.001
Partnered	378 (88.5)	523 (81.1)	300 (67.9)		1,021 (89.0)	896 (83.3)	520 (73.9)	
Single	49 (11.5)	122 (18.9)	142 (32.1)		126 (11.0)	180 (16.7)	184 (26.1)	
Residence status, *N* (%)				<0.001				<0.001
Rural	329 (77.0)	603 (93.5)	422 (95.5)		745 (65.0)	921 (85.6)	641 (91.1)	
Urban	98 (23.0)	42 (6.5)	20 (4.5)		402 (35.0)	155 (14.4)	63 (8.9)	
Geographic distribution, *N* (%)				<0.001				<0.001
Eastern China	163 (38.2)	189 (29.3)	134 (30.3)		485 (42.3)	434 (40.3)	270 (38.4)	
Central China	176 (41.2)	243 (37.7)	145 (32.8)		448 (39.1)	360 (33.5)	235 (33.4)	
Western China	88 (20.6)	213 (33.0)	163 (36.9)		214 (18.7)	282 (26.2)	199 (28.3)	
Household family income per year (RMB), *N* (%)				<0.001				<0.001
0–9,999	244 (57.1)	454 (70.4)	310 (70.1)		510 (44.5)	627 (58.3)	469 (66.6)	
10,000–49,999	152 (35.6)	159 (24.7)	109 (24.7)		508 (44.3)	362 (33.6)	177 (25.1)	
50,000–	31 (7.3)	32 (5.0)	23 (5.2)		129 (11.2)	87 (8.1)	58 (8.2)	
Medical insurance, *N* (%)				<0.001				<0.001
Urban and Rural Resident Basic Medical Insurance	334 (78.2)	583 (90.4)	403 (91.2)		740 (64.5)	904 (84.0)	613 (87.1)	
Urban Employee Basic Medical Insurance	59 (13.8)	16 (2.5)	3 (0.7)		267 (23.3)	74 (6.9)	17 (2.4)	
Others	34 (8.0)	46 (7.1)	36 (8.1)		140 (12.2)	98 (9.1)	74 (10.5)	

Sensitivity analyses showed that when examining the factors influencing physical disability trajectories (Supplementary Table S1), cognitive function score was a risk factor for both the MIDDLE group (OR: 1.24; 95:CI: 1.14, 1.34) and HIGH group (1.67; 1.50, 1.85), compared to LOW disability group. No significant interaction effect of cognitive function score with any characteristics was founded. Baseline physical disability score was not associated with cognitive trajectories (Supplementary Table S2), although the interaction of medical insurance and physical disability score was significant. Fourteen types of available comorbidities are summarized in Supplementary Table S3. Hypertension was associated with high cognitive trajectory group (1.58; 1.22, 2.05), compared to low group, and might also interact with education and residence status (Supplementary Table S4) to affect cognitive function trajectory. Regarding physical disability trajectory, hypertension was associated with both middle (1.84; 1.55, 2.19) and high disability trajectory groups (2.08; 1.46, 2.95), but no significant interaction of hypertension with other characteristics was observed (Supplementary Table S5).

## Discussion

Based on a large prospective cohort in China, three trajectories for cognitive function and physical disability were identified in people aged 60 and above. Compared with those free of disability at baseline, a larger proportion of older people with disability at baseline showed rapid cognitive deterioration. Furthermore, we found that the trajectories of cognitive function and disability shared mostly the same contributing factors.

The development trajectories of cognitive function in the older Chinese people included in this study were categorized into LOW, MIDDLE, and HIGH groups, representing groups with stable good cognitive function, slightly worse cognitive function with dramatic progression, and the lowest level of cognitive function at baseline followed by rapid progression, respectively. These group features were consistent with the findings of Su et al. in China, in a study that was also based on CHARLS data and applied GBTM (2022) (21), Casanova et al. in the UK (2020) (61), and Hamilton et al. in the US (2021) (22), suggesting that older people from different countries may display similar patterns of progression in cognitive degeneration. However, we did not find a reversal trajectory as observed by Summers et al. in an Australian population, in which 24.7% of people aged over 60 with mild cognitive impairment recovered to a level of unimpaired cognitive function over the following 20 months (62). Another study by Ye et al., based on the Chinese Longitudinal Healthy Longevity Survey cohort of Chinese people aged 65 and above and spanning 12 years, identified a group with a slight improvement in cognitive function over time. This group, accounting for 19.16% of the population, showed moderate levels of cognitive function at baseline and a slight increase in cognitive function by one point (20). The differences in the results of these studies may stem from variation in population characteristics, measurement tools, medical services, and other factors.

Because the causes of disability in older people are complex, the number and shape of disability trajectories have varied widely in previous studies (25, 26, 28, 63, 64). Nevertheless, regardless of the classification model, the disability trajectories of Chinese older people have consistently been categorized into three groups (24, 30, 31, 65–68). Most studies have shown that these groups exhibit the characteristics of a low disability level remaining consistent, a low baseline disability level followed by rapid development, and a high disability level with rapid development, respectively (24, 30, 65). These findings are in line with the trajectories identified in our study.

To the best of our knowledge, this is the first Chinese study to consider both disability and cognitive function together, which is of great significance in shedding light on co-morbidity in the older people. We found that the cognitive function levels in older people were characterized by the same three trajectories regardless of the presence or absence of physical disability at baseline. However, a greater proportion of the older individuals who had limitations in daily activities at baseline showed rapid declines in cognitive function post-baseline. These results to some extent validate the findings of Verlinden et al. (69), who established a link between cognitive function and disability; that is, dementia patients showed memory impairment, a decreased Mini Mental State Examination scale score, IADL restriction, and Basic Activities of Daily Living restriction in the 16 years before dementia diagnosis (69). In other words, physical disability in the older people accelerates the decline in cognitive function, which in turn worsens physical limitations.

In terms of the factors contributing to the identified trajectories of cognitive function, we found that more highly educated older people had higher baseline cognitive levels and were less likely to be on a rapid decline trajectory, which is consistent with previous findings (70–73). These results support the cognitive reserve hypothesis, which states that the brain is able to utilize available neural structures as a backup or reserve, and therefore education early in life can delay the clinical expression of dementia by influencing the brain’s pathological response. This hypothesis has been validated in animal models (74). Casanova et al. indicated that the most prominent predictor of cognitive trajectory is education level (61). Even education in later life has been shown to protect cognitive function (75). As the prevalence and incidence of dementia among the older people in China with low education levels are on the rise (76), we suggest that educational efforts targeting middle-aged and older Chinese adults with low education levels may help reduce their risk of dementia and rapid cognitive deterioration.

Our findings suggest that, to some extent, a high socio-economic status helps maintain a high level of cognitive function and physical ability. In particular, household income was only associated with the trajectory characterized by a low physical disability level at baseline followed by rapid development. As household income decreased, the probability of an older person following this trajectory increased significantly (OR_1_ = 0.765, OR_2_ = 0.634). This phenomenon is consistent with the finding of Nusselder (77) that low-income groups were more likely to follow a trajectory characterized by a sudden increase in disability, because they were at an increased risk of disabling chronic diseases due to behaviors that were not beneficial to their health. In addition, Taylor et al. found that education, while effective in preventing the onset of disability, was less effective in slowing the progression of disability at a certain level of income (47). In other words, income may play a more crucial role in preventing disability than education. Therefore, in areas with better economic status, education for the older people may delay dementia, while in economically disadvantaged areas, vigorous economic development and creating economic security for the older people would be effective in reducing the overall degree of disability.

Notably, this study is the first to find that the type of medical insurance also affects the baseline cognitive levels and rate of cognitive decline in older people. In China, medical insurance is divided into two categories: medical insurance for urban workers and medical insurance for urban and rural residents. The former mainly covers employees, and the latter covers urban and rural unemployed people and freelance workers, such as housewives and farmers. Compared with urban and rural residents with medical insurance, urban workers with medical insurance tend to have higher salaries, to enjoy more social security after retirement, and to live in better neighborhoods. In this study, the vast majority of the participants held medical insurance for urban and rural residents. They faced a greater risk of being on the trajectory characterized by the lowest baseline cognitive level and rapid cognitive decline post-baseline. Therefore, we posit that the association between the type of medical insurance and the trajectory of cognitive function exists because this essential indicator encompasses economic income, living environment, and other factors. However, this finding requires further validation through future studies.

We found that women were at a greater disadvantage than men in terms of both baseline cognitive level and rate of cognitive decline. This aligns with the findings of other studies in Chinese older people (17, 72). This association is likely to operate through the mediating effects of nutrition, education, and social participation. Due to traditional norms prevailing in China in the mid-20th century, men enjoyed a higher status in the family, prioritized access to scarce resources like education and food, and higher levels of social participation and social support. Women, in contrast, were more likely to experience malnutrition (45, 78, 79), possess a lower level of education, and face a lack of social interaction (80–82), all of which have been identified in previous studies as risk factors for dementia. Similar patterns have been observed in older people in other developing countries, such as Brazil (83).

In the current study, being partnered played a positive role in maintaining cognitive function in older people. Research has indicated that the impact of marriage on cognition is primarily explained by marital quality and the duration of widowhood. Individuals in high-quality marriages tend to receive emotional support and care from their spouses, so higher marital quality is often associated with higher cognitive function (84). Another study (2019) indicated that the converse was also true, i.e., the cognitive level of widowed older people was lower than that of their non-widowed counterparts, and the likelihood of cognitive decline increased with the duration of widowhood. However, the effects of stressful life events, such as widowhood, on people’s cognitive function were observed to be delayed, with a sharp decline in cognitive function emerging 4 to 6 years after late-life widowhood (85).

This study has some limitations. First, we identified the cognitive function trajectories of older people in disabled and non-disabled participants separately to explore the combined trajectory of physical disability and cognitive function, rather than identifying their covarying trajectory. This is due to the heterogeneity in the methods of assessing cognitive level and physical disability, with the former typically having a scoring system and the latter being assessed by the number of items of daily living in which impairments are experienced. Second, some potentially important variables were not included in the model due to their unavailability. For example, a large number of studies have confirmed that the APOE gene is a high-risk genetic factor for progression of dementia. Not adjusting for this factor may, to some extent, have biased the effect estimates of the factors that were tested.

## Conclusion

In this study, the cognitive function trajectories of Chinese older people fell into three characteristic groups: those maintaining the highest level of cognitive function, those with a moderate baseline level of cognitive function and dramatic progression, and those with the lowest baseline level of cognitive function and rapid progression. The disability trajectories also fell into three characteristic groups: a consistently low disability level, a low initial disability level with rapid development, and a high baseline disability level with rapid development. Analyzing cognitive function and physical disability together, we found that compared with those without disability at baseline, a greater proportion of older people with disability at baseline experienced rapid cognitive deterioration. In addition, education, income, type of medical insurance, gender, and marital status were found to be instrumental in the progression of disability and cognitive function impairment in the older population. The results suggest that the Chinese government, focusing on the central and western regions and rural areas, should develop education for the older people and increase their level of economic security to slow the rate of cognitive function decline and disability among this age group in China. These could become important measures to cope with population aging.

## Data availability statement

Publicly available datasets were analyzed in this study. This data can be found at: https://charls.charlsdata.com/pages/data/111/zh-cn.html.

## Ethics statement

Ethical review and approval was not required for the study on human participants in accordance with the local legislation and institutional requirements. Written informed consent from the patients/participants or the patients’/participants’ legal guardian/next of kin was not required to participate in this study in accordance with the national legislation and the institutional requirements.

## Author contributions

SC: Writing – review & editing, Conceptualization, Data curation, Formal analysis, Funding acquisition, Investigation, Methodology, Project administration, Resources, Software, Visualization, Writing – original draft. RY: Data curation, Validation, Writing – review & editing, Formal analysis, Investigation, Writing – original draft. KW: Data curation, Investigation, Methodology, Software, Visualization, Writing – review & editing, Writing – original draft. QW: Formal analysis, Methodology, Supervision, Validation, Writing – review & editing, Investigation. HZ: Project administration, Resources, Supervision, Writing – review & editing, Conceptualization. LL: Project administration, Resources, Supervision, Writing – review & editing. WC: Conceptualization, Data curation, Formal analysis, Funding acquisition, Methodology, Resources, Supervision, Writing – review & editing. LS: Conceptualization, Resources, Supervision, Writing – review & editing.
