# Supplementary material for: Trajectories and influencing factors of cognitive function and physical disability in Chinese older people
Source: Front Public Health. 2024 Jul 4;12:1380657. doi: 10.3389/fpubh.2024.1380657 (PMC11256785; doi:10.3389/fpubh.2024.1380657)
Supplement: Supplementary file 1 [file Table_1.DOCX]

Table S1. Interaction of cognitive function and baseline characteristics on disability trajectories.

| Baseline characteristics | Reference | Level | OR(95%CI) | | | |  |
| --- | --- | --- | --- | --- | --- | --- | --- |
|  |  |  | Middle vs Low | | | High vs Low |  |
| Age | 60-69 | 70-79 | 2.41 (0.94,6.18) | 3.44 (0.75,15.73) | | |  |
|  |  | 80- | **18.78 (1.84,191.91)** | **37.41 (1.13,1238.95)** | | |  |
| Sex | Male | Female | 1.16 (0.55,2.45) | 1.08 (0.27,4.35) | | |  |
| Education | No formal education | Primary school | **0.23 (0.07,0.74)** | 0.25 (0.05,1.24) | | |  |
|  |  | Junior middle school | **0.16 (0.04,0.66)** | 0.06 (0.00,1.90) | | |  |
|  |  | Middle school or above | **0.03 (0.00,0.23)** | **0.00 (0.00,5.79)** | | |  |
| Marital status | Partnered | Single | 1.09 (0.39,2.99) | 0.63 (0.11,3.56) | | |  |
| Residence status | Rural | Urban | 0.65 (0.21,1.95) | 1.90 (0.21,17.17) | | |  |
| Geographic distribution | Easten China | Central China | 0.96 (0.43,2.17) | 0.35 (0.07,1.66) | | |  |
|  |  | Western China | 1.23 (0.49,3.07) | 2.39 (0.50,11.44) | | |  |
| Household family income per year | 0-9999 | 10000-49999 | 0.72 (0.33,1.61) | 0.31 (0.07,1.41) | | |  |
|  |  | 50000- | 1.83 (0.51,6.54) | 7.16 (0.73,70.20) | | |  |
| Medical insurance | New cooperative medical insurance | Urban Employee Basic Medical Insurance | 1.05 (0.29,3.80) | 2.14 (0.07,65.11) | | |  |
|  |  | Others | 0.67 (0.18,2.56) | 0.72 (0.07,7.42) | | |  |
| Cognitive function score |  |  | **1.24 (1.14,1.34)** | | **1.67 (1.50,1.85)** | | |
| Cognitive function score *age |  | Cognitive function score*70-79 | 0.98 (0.93,1.04) | | 1.00 (0.92,1.08) | | |
|  |  | Cognitive function score*80- | 0.88 (0.77,1.02) | | 0.93 (0.77,1.11) | | |
| Cognitive function score*Sex |  | Cognitive function score *female | 0.99 (0.95,1.04) | | 1.01 (0.94,1.08) | | |
| Cognitive function score *Education |  | Cognitive function score*Primary school | 1.00 (0.93,1.08) | | 0.94 (0.86,1.02) | | |
|  |  | Cognitive function score*Junior middle school | 0.98 (0.90,1.07) | | 0.95 (0.80,1.12) | | |
|  |  | Cognitive function score*Middle school or above | 1.05 (0.93,1.19) | | 1.07 (0.74,1.55) | | |
| Cognitive function score *Marital status |  | Cognitive function score*Single | 1.00 (0.95,1.07) | | 1.04 (0.95,1.13) | | |
| Cognitive function score *Residence status |  | Cognitive function score*Urban | 1.00 (0.93,1.07) | | 0.95 (0.85,1.06) | | |
| Cognitive function score *Geographic distribution |  | Cognitive function score*Central China | 1.01 (0.96,1.06) | | 1.05 (0.97,1.14) | | |
|  |  | Cognitive function score*Western China | 1.01 (0.95,1.06) | | 0.99 (0.91,1.07) | | |
| Cognitive function score *Household family income per year |  | Cognitive function score*10000-49999 | 1.03 (0.98,1.08) | | 1.06 (0.98,1.15) | | |
|  |  | Cognitive function score*50000- | 0.97 (0.90,1.05) | | 0.93 (0.82,1.04) | | |
| Cognitive function score *Medical insurance |  | Cognitive function score* Urban Employee Basic Medical Insurance | 0.96 (0.89,1.04) | | 0.91 (0.77,1.08) | | |
|  |  | Cognitive function score* Others | 1.02 (0.94,1.11) | | 1.02 (0.90,1.14) | | |

Table S2. Interaction of disability status and baseline characteristics on cognitive trajectories.

| Baseline characteristics | Reference | Level | OR(95%CI) | | | |  |
| --- | --- | --- | --- | --- | --- | --- | --- |
|  |  |  | Middle vs Low | | | High vs Low |  |
| Age | 60-69 | 70-79 | **1.97 (1.54,2.52)** | **3.36 (2.47,4.58)** | | |  |
|  |  | 80- | **4.81 (2.00,11.55)** | **25.92 (10.02,67.05)** | | |  |
| Sex | Male | Female | 1.04 (0.85,1.27) | **1.71 (1.30,2.24)** | | |  |
| Education | No formal education | Primary school | **0.18 (0.14,0.25)** | **0.04 (0.03,0.06)** | | |  |
|  |  | Junior middle school | **0.07 (0.05,0.10)** | **0.01 (0.00,0.02)** | | |  |
|  |  | Middle school or above | **0.04 (0.02,0.06)** | **0.00 (0.00,0.01)** | | |  |
| Marital status | Partnered | Single | 1.23 (0.94,1.62) | **1.52 (1.09,2.12)** | | |  |
| Residence status | Rural | Urban | **0.61 (0.46,0.82)** | **0.39 (0.25,0.60)** | | |  |
| Geographic distribution | Easten China | Central China | 1.10 (0.89,1.36) | 1.26 (0.94,1.69) | | |  |
|  |  | Western China | **1.43 (1.13,1.82)** | **1.93 (1.40,2.65)** | | |  |
| Household family income per year | 0-9999 | 10000-49999 | 0.93 (0.76,1.14) | **0.73 (0.55,0.97)** | | |  |
|  |  | 50000- | 1.09 (0.78,1.53) | 1.22 (0.77,1.95) | | |  |
| Medical insurance | New cooperative medical insurance | Urban Employee Basic Medical Insurance | **0.55 (0.39,0.79)** | **0.36 (0.18,0.73)** | | |  |
|  |  | Others | 1.03 (0.74,1.44) | **1.55 (1.01,2.40)** | | |  |
| Disability status | Nondisabled | Disabled | 1.04 (0.54,2.01) | | 1.63 (0.79,3.36) | | |
| Disability status *age |  | Disabled*70-79 | 1.37 (0.84,2.25) | | **2.03 (1.16,3.56)** | | |
|  |  | Disabled*80- | 0.35 (0.09,1.38) | | 0.39 (0.09,1.61) | | |
| Disability status*Sex |  | Yes*female | 1.02 (0.70,1.50) | | 0.90 (0.56,1.45) | | |
| Disability status *Education |  | Disabled*Primary school | 1.22 (0.68,2.17) | | 0.97 (0.53,1.78) | | |
|  |  | Disabled*Junior middle school | 0.92 (0.45,1.90) | | 0.77 (0.24,2.49) | | |
|  |  | Disabled*Middle school or above | 1.45 (0.50,4.19) | | 1.17 (0.09,15.64) | | |
| Disability status *Marital status |  | Disabled*Single | 1.12 (0.66,1.93) | | 1.13 (0.61,2.06) | | |
| Disability status *Residence status |  | Disabled*Urban | **0.54 (0.30,0.97)** | | 0.50 (0.22,1.12) | | |
| Disability status *Geographic distribution |  | Disabled*Central China | 1.50 (0.99,2.26) | | 1.45 (0.87,2.44) | | |
|  |  | Disabled*Western China | 1.21 (0.76,1.93) | | 1.70 (0.98,2.98) | | |
| Disability status *Household family income per year |  | Disabled*10000-49999 | 1.31 (0.87,1.95) | | 1.21 (0.73,2.00) | | |
|  |  | Disabled*50000- | 0.94 (0.44,2.02) | | 0.95 (0.38,2.41) | | |
| Disability status *Medical insurance |  | Disabled* Urban Employee Basic Medical Insurance | 1.19 (0.54,2.59) | | 0.99 (0.24,4.10) | | |
|  |  | Disabled* Others | 0.97 (0.50,1.91) | | 0.55 (0.24,1.24) | | |

Table S3. Baseline comorbidity of the participants

|  | Level | Overall | Cognitive trajectory | | | | Disability trajectory | | | | |  |
| --- | --- | --- | --- | --- | --- | --- | --- | --- | --- | --- | --- | --- |
|  |  |  | low | middle | high | *P* | | low | middle | high | *P* | |
| N |  | 4441 | 1546 | 1631 | 1264 |  | | 2341 | 1774 | 326 |  | |
| Hypertension (%) | No | 3109 (70.0) | 1055 (68.2) | 1162 (71.2) | 892 (70.6) | 0.066 | | 1719 (73.4) | 1200 (67.6) | 190 (58.3) | **<0.001** | |
|  | Yes | 1311 (29.5) | 488 (31.6) | 460 (28.2) | 363 (28.7) |  | | 616 (26.3) | 561 (31.6) | 134 (41.1) |  | |
|  | Missing | 21 (0.5) | 3 (0.2) | 9 (0.6) | 9 (0.7) |  | | 6 (0.3) | 13 (0.7) | 2 (0.6) |  | |
| Dyslipidemia (%) | No | 3896 (87.7) | 1305 (84.4) | 1446 (88.7) | 1145 (90.6) | **<0.001** | | 2071 (88.5) | 1552 (87.5) | 273 (83.7) | **0.002** | |
|  | Yes | 464 (10.4) | 224 (14.5) | 153 (9.4) | 87 (6.9) |  | | 242 (10.3) | 176 (9.9) | 46 (14.1) |  | |
|  | Missing | 81 (1.8) | 17 (1.1) | 32 (2.0) | 32 (2.5) |  | | 28 (1.2) | 46 (2.6) | 7 (2.1) |  | |
| Diabetes or high blood sugar (%) | No | 4127 (92.9) | 1405 (90.9) | 1523 (93.4) | 1199 (94.9) | **<0.001** | | 2195 (93.8) | 1643 (92.6) | 289 (88.7) | **0.012** | |
|  | Yes | 277 (6.2) | 131 (8.5) | 94 (5.8) | 52 (4.1) |  | | 129 (5.5) | 114 (6.4) | 34 (10.4) |  | |
|  | Missing | 37 (0.8) | 10 (0.6) | 14 (0.9) | 13 (1.0) |  | | 17 (0.7) | 17 (1.0) | 3 (0.9) |  | |
| Cancer or malignt tumor (%) | No | 4381 (98.6) | 1522 (98.4) | 1613 (98.9) | 1246 (98.6) | 0.808 | | 2312 (98.8) | 1747 (98.5) | 322 (98.8) | 0.91 | |
|  | Yes | 37 (0.8) | 14 (0.9) | 11 (0.7) | 12 (0.9) |  | | 18 (0.8) | 16 (0.9) | 3 (0.9) |  | |
|  | Missing | 23 (0.5) | 10 (0.6) | 7 (0.4) | 6 (0.5) |  | | 11 (0.5) | 11 (0.6) | 1 (0.3) |  | |
| Chronic lung diseases (%) | No | 3863 (87.0) | 1345 (87.0) | 1396 (85.6) | 1122 (88.8) | 0.074 | | 2110 (90.1) | 1490 (84.0) | 263 (80.7) | **<0.001** | |
|  | Yes | 560 (12.6) | 197 (12.7) | 228 (14.0) | 135 (10.7) |  | | 224 (9.6) | 276 (15.6) | 60 (18.4) |  | |
|  | Missing | 18 (0.4) | 4 (0.3) | 7 (0.4) | 7 (0.6) |  | | 7 (0.3) | 8 (0.5) | 3 (0.9) |  | |
| Liver disease (%) | No | 4236 (95.4) | 1458 (94.3) | 1562 (95.8) | 1216 (96.2) | **0.007** | | 2252 (96.2) | 1678 (94.6) | 306 (93.9) | **0.019** | |
|  | Yes | 173 (3.9) | 81 (5.2) | 54 (3.3) | 38 (3.0) |  | | 80 (3.4) | 76 (4.3) | 17 (5.2) |  | |
|  | Missing | 32 (0.7) | 7 (0.5) | 15 (0.9) | 10 (0.8) |  | | 9 (0.4) | 20 (1.1) | 3 (0.9) |  | |
| Heart problems (%) | No | 3752 (84.5) | 1261 (81.6) | 1400 (85.8) | 1091 (86.3) | **<0.001** | | 2050 (87.6) | 1452 (81.8) | 250 (76.7) | **<0.001** | |
|  | Yes | 658 (14.8) | 278 (18.0) | 219 (13.4) | 161 (12.7) |  | | 279 (11.9) | 307 (17.3) | 72 (22.1) |  | |
|  | Missing | 31 (0.7) | 7 (0.5) | 12 (0.7) | 12 (0.9) |  | | 12 (0.5) | 15 (0.8) | 4 (1.2) |  | |
| Stroke (%) | No | 4309 (97.0) | 1501 (97.1) | 1588 (97.4) | 1220 (96.5) | 0.106 | | 2310 (98.7) | 1710 (96.4) | 289 (88.7) | **<0.001** | |
|  | Yes | 119 (2.7) | 43 (2.8) | 40 (2.5) | 36 (2.8) |  | | 26 (1.1) | 58 (3.3) | 35 (10.7) |  | |
|  | Missing | 13 (0.3) | 2 (0.1) | 3 (0.2) | 8 (0.6) |  | | 5 (0.2) | 6 (0.3) | 2 (0.6) |  | |
| Kidney disease (%) | No | 4126 (92.9) | 1436 (92.9) | 1504 (92.2) | 1186 (93.8) | 0.056 | | 2207 (94.3) | 1631 (91.9) | 288 (88.3) | **<0.001** | |
|  | Yes | 288 (6.5) | 105 (6.8) | 117 (7.2) | 66 (5.2) |  | | 124 (5.3) | 131 (7.4) | 33 (10.1) |  | |
|  | Missing | 27 (0.6) | 5 (0.3) | 10 (0.6) | 12 (0.9) |  | | 10 (0.4) | 12 (0.7) | 5 (1.5) |  | |
| Stomach or other digestive disease (%) | No | 3359 (75.6) | 1207 (78.1) | 1210 (74.2) | 942 (74.5) | 0.058 | | 1871 (79.9) | 1271 (71.6) | 217 (66.6) | **<0.001** | |
|  | Yes | 1071 (24.1) | 336 (21.7) | 418 (25.6) | 317 (25.1) |  | | 467 (19.9) | 496 (28.0) | 108 (33.1) |  | |
|  | Missing | 11 (0.2) | 3 (0.2) | 3 (0.2) | 5 (0.4) |  | | 3 (0.1) | 7 (0.4) | 1 (0.3) |  | |
| Emotiol, nervous, or psychiatric problems (%) | No | 4355 (98.1) | 1523 (98.5) | 1599 (98.0) | 1233 (97.5) | 0.158 | | 2307 (98.5) | 1727 (97.4) | 321 (98.5) | **0.011** | |
|  | Yes | 62 (1.4) | 18 (1.2) | 25 (1.5) | 19 (1.5) |  | | 20 (0.9) | 37 (2.1) | 5 (1.5) |  | |
|  | Missing | 24 (0.5) | 5 (0.3) | 7 (0.4) | 12 (0.9) |  | | 14 (0.6) | 10 (0.6) | 0 (0.0) |  | |
| Memory-related disease (%) | No | 4346 (97.9) | 1519 (98.3) | 1596 (97.9) | 1231 (97.4) | 0.332 | | 2314 (98.8) | 1726 (97.3) | 306 (93.9) | **<0.001** | |
|  | yes | 78 (1.8) | 22 (1.4) | 31 (1.9) | 25 (2.0) |  | | 21 (0.9) | 38 (2.1) | 19 (5.8) |  | |
|  | missing | 17 (0.4) | 5 (0.3) | 4 (0.2) | 8 (0.6) |  | | 6 (0.3) | 10 (0.6) | 1 (0.3) |  | |
| Arthritis or rheumatism (%) | no | 2700 (60.8) | 1005 (65.0) | 989 (60.6) | 706 (55.9) | **<0.001** | | 1644 (70.2) | 927 (52.3) | 129 (39.6) | **<0.001** | |
|  | yes | 1731 (39.0) | 538 (34.8) | 639 (39.2) | 554 (43.8) |  | | 694 (29.6) | 842 (47.5) | 195 (59.8) |  | |
|  | missing | 10 (0.2) | 3 (0.2) | 3 (0.2) | 4 (0.3) |  | | 3 (0.1) | 5 (0.3) | 2 (0.6) |  | |
| Asthma (%) | no | 4217 (95.0) | 1464 (94.7) | 1541 (94.5) | 1212 (95.9) | 0.232 | | 2256 (96.4) | 1663 (93.7) | 298 (91.4) | **<0.001** | |
|  | yes | 208 (4.7) | 76 (4.9) | 86 (5.3) | 46 (3.6) |  | | 76 (3.2) | 105 (5.9) | 27 (8.3) |  | |
|  | missing | 16 (0.4) | 6 (0.4) | 4 (0.2) | 6 (0.5) |  | | 9 (0.4) | 6 (0.3) | 1 (0.3) |  | |

Table S4. Factors associated with cognitive function trajectories after including hypertension and the interaction with other characteristics

| Baseline characteristics | Reference | Level | OR(95%CI) | | | |  |
| --- | --- | --- | --- | --- | --- | --- | --- |
|  |  |  | Middle vs Low | | | High vs Low |  |
| Age | 60-69 | 70-79 | **2.12 (1.64,2.74)** | **4.18 (3.08,5.66)** | | |  |
|  |  | 80- | **4.07 (1.61,10.28)** | **27.61 (10.56,72.16)** | | |  |
| Sex | Male | Female | 0.74 (0.40,1.38) | **0.46 (0.22,0.94)** | | |  |
| Education | No formal education | Primary school | **0.17 (0.13,0.24)** | **0.03 (0.02,0.05)** | | |  |
|  |  | Junior middle school | **0.06 (0.04,0.09)** | **0.00 (0.00,0.01)** | | |  |
|  |  | Middle school or above | **0.03 (0.02,0.06)** | **0.00 (0.00,0.01)** | | |  |
| Marital status | Partnered | Single | **1.37 (1.03,1.82)** | **1.60 (1.15,2.25)** | | |  |
| Residence status | Rural | Urban | **0.58 (0.43,0.79)** | **0.42 (0.27,0.66)** | | |  |
| Geographic distribution | Easten China | Central China | **1.28 (1.03,1.59)** | **1.39 (1.04,1.85)** | | |  |
|  |  | Western China | **1.58 (1.23,2.01)** | **2.69 (1.98,3.65)** | | |  |
| Household family income per year | 0-9999 | 10000-49999 | 0.97 (0.79,1.19) | **0.67 (0.51,0.88)** | | |  |
|  |  | 50000- | 0.95 (0.66,1.37) | 1.16 (0.72,1.87) | | |  |
| Medical insurance | New cooperative medical insurance | Urban Employee Basic Medical Insurance | **0.58 (0.39,0.85)** | **0.29 (0.13,0.64)** | | |  |
|  |  | Others | 1.06 (0.76,1.49) | 1.12 (0.73,1.73) | | |  |
| Hypertension | No | Yes | 1.09 (0.89,1.33) | | **1.58 (1.22,2.05)** | | |
| Hypertension *age |  | Yes*70-79 | 1.05 (0.66,1.65) | | 1.28 (0.74,2.19) | | |
|  |  | Yes*80- | 0.73 (0.19,2.86) | | 0.43 (0.10,1.85) | | |
| Hypertension*Sex |  | Yes*female | 1.01 (0.70,1.47) | | 1.37 (0.84,2.23) | | |
| Hypertension *Education |  | Yes*Primary school | 1.38 (0.80,2.39) | | **1.95 (1.08,3.50)** | | |
|  |  | Yes*Junior middle school | 1.28 (0.64,2.57) | | **3.26 (1.05,10.10)** | | |
|  |  | Yes*Middle school or above | 2.09 (0.80,5.43) | | **0.00 (0.00,0.00)** | | |
| Hypertension *Marital status |  | Yes*Single | 0.70 (0.43,1.17) | | 0.94 (0.52,1.67) | | |
| Hypertension *Residence status |  | Yes*Urban | 0.70 (0.41,1.19) | | **0.44 (0.20,0.95)** | | |
| Hypertension *Geographic distribution |  | Yes*Central China | 0.97 (0.66,1.45) | | 1.16 (0.69,1.93) | | |
|  |  | Yes*Western China | 0.91 (0.58,1.44) | | 0.72 (0.41,1.27) | | |
| Hypertension *Household family income per year |  | Yes*10000-49999 | 1.07 (0.72,1.58) | | 1.44 (0.87,2.38) | | |
|  |  | Yes*50000- | 1.39 (0.73,2.64) | | 0.98 (0.41,2.34) | | |
| Hypertension *Medical insurance |  | Yes* Urban Employee Basic Medical Insurance | 0.98 (0.50,1.92) | | 1.36 (0.39,4.79) | | |
|  |  | Yes* Others | 0.95 (0.50,1.79) | | 1.40 (0.63,3.14) | | |

Table S5. Factors associated with physical disability trajectories after including hypertension and the interaction with other characteristics

| Baseline characteristics | Reference | Level | OR(95%CI) | | | |  |
| --- | --- | --- | --- | --- | --- | --- | --- |
|  |  |  | Middle vs Low | | | High vs Low |  |
| Age | 60-69 | 70-79 | **2.06 (1.70,2.51)** | **3.52 (2.48,5.00)** | | |  |
|  |  | 80- | **3.68 (2.26,5.99)** | **8.81 (4.41,17.60)** | | |  |
| Sex | Male | Female | 1.44 (0.92,2.25) | **2.66 (1.18,6.03)** | | |  |
| Education | No formal education | Primary school | **0.56 (0.47,0.67)** | **0.49 (0.35,0.70)** | | |  |
|  |  | Junior middle school | **0.35 (0.25,0.47)** | **0.28 (0.14,0.58)** | | |  |
|  |  | Middle school or above | **0.41 (0.27,0.64)** | **0.12 (0.03,0.54)** | | |  |
| Marital status | Partnered | Single | 1.23 (0.99,1.52) | 1.09 (0.73,1.61) | | |  |
| Residence status | Rural | Urban | **0.71 (0.54,0.94)** | 0.68 (0.38,1.22) | | |  |
| Geographic distribution | Easten China | Central China | **1.70 (1.41,2.06)** | **2.56 (1.72,3.81)** | | |  |
|  |  | Western China | **1.77 (1.44,2.16)** | **2.92 (1.94,4.40)** | | |  |
| Household family income per year | 0-9999 | 10000-49999 | **0.78 (0.66,0.94)** | 0.88 (0.62,1.25) | | |  |
|  |  | 50000- | **0.69 (0.50,0.95)** | 0.52 (0.24,1.11) | | |  |
| Medical insurance | New cooperative medical insurance | Urban Employee Basic Medical Insurance | 0.73 (0.50,1.06) | 0.74 (0.31,1.79) | | |  |
|  |  | Others | 1.09 (0.82,1.45) | 1.14 (0.66,1.97) | | |  |
| Hypertension | No | Yes | **1.84 (1.55,2.19)** | | **2.08 (1.46,2.95)** | | |
| Hypertension *age |  | Yes*70-79 | 1.03 (0.72,1.47) | | 0.86 (0.49,1.51) | | |
|  |  | Yes*80- | 1.21 (0.47,3.12) | | 0.92 (0.26,3.23) | | |
| Hypertension*Sex |  | Yes*female | 1.07 (0.77,1.48) | | 0.87 (0.49,1.54) | | |
| Hypertension *Education |  | Yes*Primary school | 1.12 (0.79,1.59) | | 1.09 (0.61,1.96) | | |
|  |  | Yes*Junior middle school | 1.19 (0.69,2.07) | | 1.60 (0.57,4.52) | | |
|  |  | Yes*Middle school or above | 1.23 (0.57,2.63) | | 4.60 (0.72,29.21) | | |
| Hypertension *Marital status |  | Yes*Single | 0.99 (0.67,1.47) | | 0.95 (0.50,1.80) | | |
| Hypertension *Residence status |  | Yes*Urban | 0.71 (0.43,1.15) | | 0.85 (0.35,2.05) | | |
| Hypertension *Geographic distribution |  | Yes*Central China | 0.92 (0.66,1.30) | | 1.18 (0.63,2.21) | | |
|  |  | Yes*Western China | 0.96 (0.65,1.42) | | 0.99 (0.50,1.95) | | |
| Hypertension *Household family income per year |  | Yes*10000-49999 | 0.92 (0.66,1.30) | | 0.70 (0.39,1.26) | | |
|  |  | Yes*50000- | 0.77 (0.44,1.36) | | 0.72 (0.22,2.36) | | |
| Hypertension *Medical insurance |  | Yes* Urban Employee Basic Medical Insurance | 1.20 (0.64,2.27) | | 0.74 (0.20,2.69) | | |
|  |  | Yes* Others | 0.72 (0.42,1.25) | | 0.59 (0.23,1.51) | | |
